# All Structures Great and Small: Nanoscale Modulations in Nematic Liquid Crystals

**DOI:** 10.3390/nano12010093

**Published:** 2021-12-29

**Authors:** Edward T. Samulski, Denisse Reyes-Arango, Alexandros G. Vanakaras, Demetri J. Photinos

**Affiliations:** 1 Department of Chemistry, University of North Carolina, Chapel Hill, NC 27599-3290, USA; 2 Department of Materials Science, University of Patras, 26504 Patras, Greece; dreyesa443@gmail.com (D.R.-A.); a.g.vanakaras@upatras.gr (A.G.V.); photinos@upatras.gr (D.J.P.)

**Keywords:** nematic dimers, polar twisted nematic, twist bend nematic, bent-core liquid crystal, nematic-nematic phase transition

## Abstract

The nature of the nanoscale structural organization in modulated nematic phases formed by molecules having a nonlinear molecular architecture is a central issue in contemporary liquid crystal research. Nevertheless, the elucidation of the molecular organization is incomplete and poorly understood. One attempt to explain nanoscale phenomena merely “shrinks down” established macroscopic continuum elasticity modeling. That explanation initially (and mistakenly) identified the low temperature nematic phase (*N_X_*), first observed in symmetric mesogenic dimers of the CB-*n*-CB series with an odd number of methylene spacers (*n*), as a twist–bend nematic (*N_TB_*). We show that the *N_X_* is unrelated to any of the elastic deformations (bend, splay, twist) stipulated by the continuum elasticity theory of nematics. Results from molecular theory and computer simulations are used to illuminate the local symmetry and physical origins of the nanoscale modulations in the *N_X_* phase, a spontaneously chiral and locally polar nematic. We emphasize and contrast the differences between the *N_X_* and theoretically conceivable nematics exhibiting spontaneous modulations of the elastic modes by presenting a coherent formulation of one-dimensionally modulated nematics based on the Frank–Oseen elasticity theory. The conditions for the appearance of nematic phases presenting true elastic modulations of the twist–bend, splay–bend, etc., combinations are discussed and shown to clearly exclude identifications with the nanoscale-modulated nematics observed experimentally, e.g., the *N_X_* phase. The latter modulation derives from packing constraints associated with nonlinear molecules—a chiral, locally-polar structural organization indicative of a new type of nematic phase.

## 1. Introduction

Unexpectedly, nonlinear (“bent-core”) dimer molecules exhibit two distinct nematic phases. The lower-temperature nematic, initially termed *N_X_* [1], exhibits form chirality—distinct macroscopic domains with left- or right-handed twisted molecular organization forming a very tight helical modulation (~10 nm)—despite the achirality of the ~3 nm-long dimers. This is one example of a category of mirror-symmetry-breaking phenomena identified in liquids and liquid crystals (LCs) [2,3].

In recent years there have been impressive developments in the synthesis and characterization of a large number of mesogens that combine molecular flexibility with a bent-core shape while exhibiting nematic phases with nanoscale modulations of the orientational order [4,5,6,7,8,9,10,11,12,13]. In attempts to rationalize this nano-scale modulation, some authors simply “miniaturized” known continuum macrostructures insinuating that the twisted structure in the *N_X_* is driven by nematic elasticity (a brief review can be found in ref. [14]). The idea of spontaneously modulated nematics originated five-decades ago [15] when R.B. Meyer proposed a “twist–bend” nematic, *N_TB_*, in which the *uniaxial and apolar director* **n** twists about a macroscopic axis generating a 1-D modulated phase wherein the ordering is locally uniaxial and apolar ( $D_{\infty h}$ ), and the modulation length scale of **n** is *macroscopic*, as implied by the elastic origin of the modulation in a continuum description of LCs.

Forcing the applicability of continuum elasticity theory on the molecular scale constitutes a grave conflation of structure scales that renders the concept of the nematic director—its symmetries and canonical distortions—ill-defined, if not totally wrong. Moreover, it obscures the underlying physics responsible for the modulation. On the other hand, both molecular theory [16] and simulations [17,18,19,20] suggest that *N_X_* has a single *polar* director **m** which is a *C*_2_ symmetry axis that roto-translates, generating a molecular length-scale 1-D modulation which confers form-chirality to the microstructure, i.e., it implies a new type of nematic organization, the so-called polar-twisted nematic, *N_PT_*.

In this work we consider the nanoscale modulation observed in the *N_X_*, focusing on the underlying physics of the molecular organization and the symmetries thereof. Results from molecular theory and simulations are used to highlight the distinguishing features of this new nematic phase and its successful description within the framework of the *N_PT_* model. In order to emphasize that the new phase is not an elastically modulated state of the conventional uniaxial nematic phase—*N_TB_* or otherwise—we present a systematic description of the 1-D spontaneously modulated states that derive from the classical Frank–Oseen theory of elasticity [21,22], stressing the conditions for the applicability of the latter and contrasting the properties of the so-derived elastically modulated states with well-established properties of the *N_X_*.

Section 2 deals with the 1-D elastically modulated nematics. The molecular theory of nanoscale-modulated nematics, leading to the *N_PT_* model is outlined in Section 3. Results from molecular dynamics simulations of model CB-*7*-CB dimers, supporting the *N_PT_* picture, are presented in Section 4. Section 5 is devoted to a critical discussion of issues regarding various models that have been proposed for the *N_X_* phase; the conclusions are drawn in Section 6.

**Terminology:** For the clarity of the exposition to follow it is useful to stress some distinctions regarding the terminology used for the various nematic phases and states involved:

- The twist–bend nematic (*N_TB_*): This is a *theoretical model*, first formulated by R.B. Meyer [15] on the basis of nematic elasticity. According to the model, states exhibiting spontaneous twist and bend *elastic deformations* can be stabilized, under certain conditions, in a uniaxial nematic phase (*N_U_*).
- The splay–bend nematic (*N_SB_*): Another *theoretical model*, also first presented by R.B. Meyer, simultaneously with the *N_TB_* [15]. Here the possibility of a *N_U_* developing stable states with spontaneous splay and bend *elastic deformations* is considered. The necessity for the formation of domains in the *N_SB_* model was pointed out in the original work.
- The *N_X_* phase: The name is reserved for the *experimentally-identified*, low- temperature nematic phase, first found in certain classes of achiral, mesogenic dimers [1] and subsequently in a variety of compounds combining mesogenic features with an overall bent-core (banana-shaped) molecular architecture, see for instance refs [8,23]. For the last decade this phase has often been termed *N_TB_*, although its experimentally determined nanoscale modulation features clearly could not stem from twist and/or bend elastic deformations of a uniaxial nematic medium as originally envisaged by R.B. Meyer [15].
- The polar-twisted nematic (*N_PT_*): A *theoretical model* formulated on the basis of molecular interactions [16,17] and presenting the possibility of local polar packing of the molecules along a polar director **m** which, in turn, undergoes periodic roto-translation modulations over molecular length scales, thereby eliminating macroscopic polarity. This model has been proposed for the description of the experimental *N_X_* phase [16].

## 2. Elastic Deformations of Uniaxial Apolar Nematics

### 2.1. Fundamental Deformations and Free Energy

The uniaxial nematic (*N_U_*) has the simplest supramolecular organization: As in ordinary liquids, there is no positional order in the *N_U_*. However, the molecules have long-range orientational order defining the *N_U_*’s “nematic director” **n**, an *apolar* local axis of complete rotational symmetry ( $D_{\infty h}$ ) [21]. This subtle orientational order imparts macroscopic anisotropy (e.g., optical, dielectric, magnetic, mechanical) that is exploited in the ubiquitous LCD because field-induced elastic deformations of **n** reversibly recover. 

Oseen and Zocher used continuum mechanics to describe such macroscopic deformations [24,25]. The continuum description of matter—viewed as an idealized medium endowed with physical properties that are devoid of microscopic structure—has its origins in a two-century-old oral presentation by Augustin Cauchy [26]. The nematic continuum approach of Oseen and Zocher was critically reviewed by Frank in the late 1950s [27]; the approach is now referred to as the Frank– Oseen, continuum theory of LCs. 

**Figure 1:**
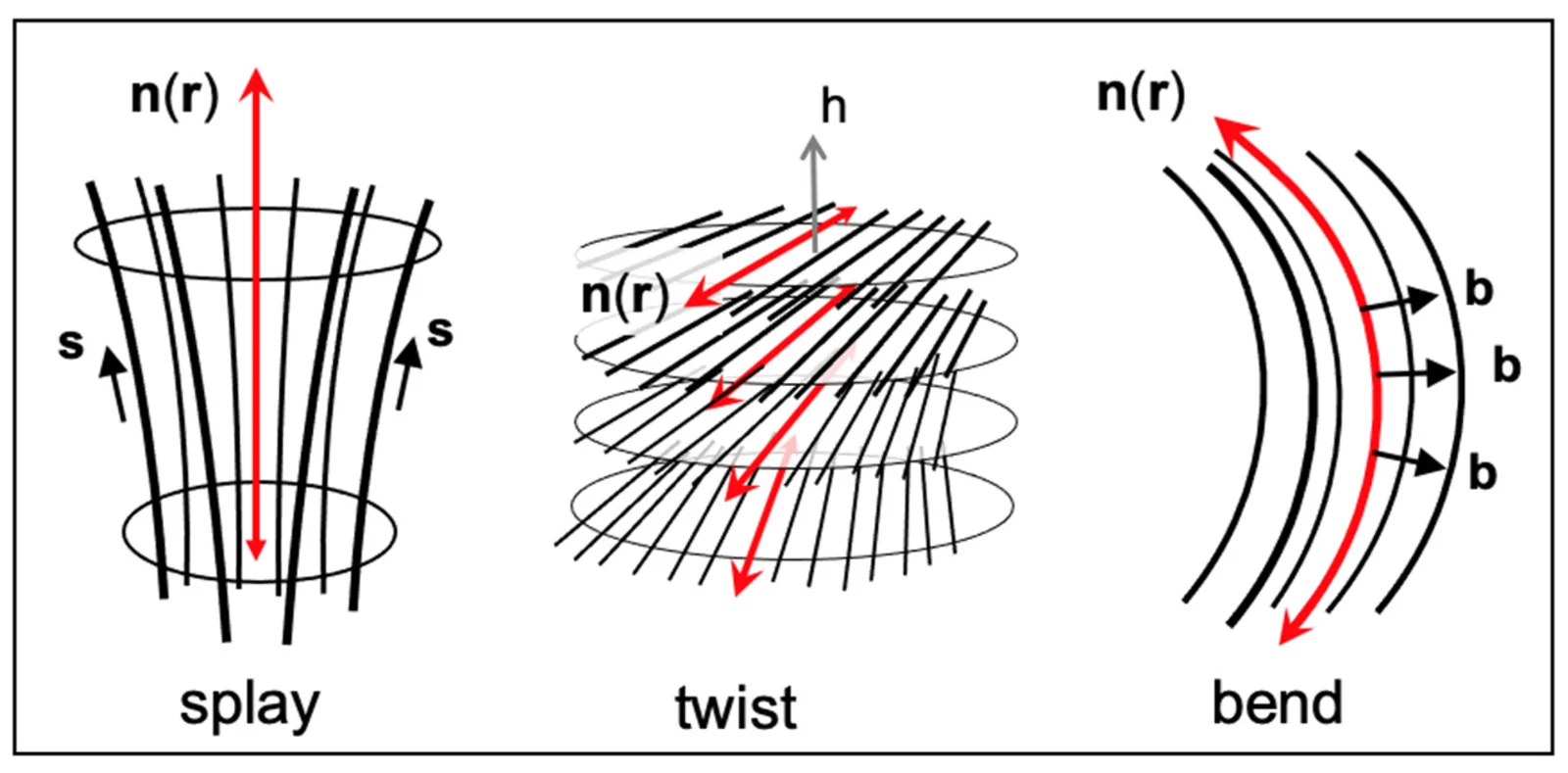
Three canonical bulk deformations of the nematic director field. In the Frank–Oseen continuum description, these deformations span macroscopic length scales. Splay (**s**) and bend (**b**) vectors are shown respectively, tangentially and radially with respect to the director field lines. The director field **n**(**r**) rotates about h in the twist distortion; double headed red arrows emphasize the apolarity of the nematic director **n**.

The Frank–Oseen theory (FOt) accounts for spatial deformations of the nematic director field, **n**(**r**), by identifying three fundamental bulk distortions of **n**(**r**) that contribute to the total free energy density. These distortions, called splay, twist and bend (Figure 1), span macroscopic scales (typically μm) and define the splay vector s=n(∇·n) , the twist pseudoscalar t=n·(∇×n) and the bend vector b=n×(∇×n) . The quadratic contributions of these distortions to the bulk deformation free energy density *F_d_* define three elastic constants, *K*_11_, *K*_22_ and *K*_33_,

(1) $$F_{d}=\frac{1}{2}K_{11}\left(s\cdot s\right)+\frac{1}{2}K_{22}t^{2}+\frac{1}{2}K_{33}\left(b\cdot b\right).$$

One can infer the delicate nature of the forces involved in deforming the **n-**field from the magnitudes of the elastic constants, typically on the order 10^−8^ Pa [28]; the elastic constant (Youngs modulus) of steel is nearly twenty orders of magnitude larger (2 × 10^11^ Pa). 

Equation (1) is strictly valid for *N_U_* nematics; different elastic contributions emerge in phases having lower point symmetries. This expression for the bulk elastic free energy density is often supplemented by two “surface terms” (see ref [22]), namely $\frac{1}{2}K_{24}\nabla \cdot \left(s+b\right)$ , representing the so called “saddle splay” deformation, and $\frac{1}{2}K_{31}\nabla \cdot \left(s-b\right).$ In addition to free energy contributions from the application of external stimuli (electric or magnetic fields, surface anchoring, etc., which will not be considered here) the free energy also receives contributions from the coupling between polar molecular attributes to the bend and splay elastic deformations. Thus, the local transverse molecular polarization density $P_{⊥}$ [15] couples to the local bend deformation b and contributes a transverse polarization component $F_{P_{⊥}}=\frac{1}{2}\alpha_{3}P_{⊥}\cdot P_{⊥}-a_{3}P_{⊥}\cdot b$ to the free energy density. Here the constant $\alpha_{3}$ accounts for the entropic increase of the free energy due to the emergence of polarization as well as for related intermolecular interactions, while the transverse flexopolarization coefficient $a_{3}$ measures the strength of the coupling between $P_{⊥}$ and b . Minimization of this part of the free energy density with respect to $P_{⊥}$ yields for the equilibrium $P_{⊥}$ the relation $P_{⊥}=\frac{a_{3}}{\alpha_{3}}b$ and for the respective minimized free energy density $F_{P_{⊥}}=-\left(\frac{1}{2}\frac{a_{3}^{2}}{\alpha_{3}}\right)\left(b\cdot b\right)$ . Therefore, inclusion of this contribution in the total free energy density amounts, according to Equation (1), to a renormalization of the bend elastic constant according to $K_{33}\Rightarrow {K^{\prime }}_{33}=\left(K_{33}-\frac{a_{3}^{2}}{\alpha_{3}}\right)$ . Similarly, the longitudinal molecular polarization density $P_{∥}$ , which couples to the splay deformation s through the flexopolarization coefficient $a_{1}$ , leads to an analogous renormalization of the splay elastic constant $K_{11}\Rightarrow {K^{\prime }}_{11}=\left(K_{11}-\frac{a_{1}^{2}}{\alpha_{1}}\right)$ . 

### 2.2. Conditions for the Applicability of the Frank–Oseen Theory of Elasticity

The FOt, from which twist, bend and splay immerge as the three fundamental deformations of a uniaxial nematic continuum, is based on a number of assumptions regarding the local symmetry of the nematic medium and the length-scale of the director-field deformations. These are listed below (see for example [21]):

(i) The deformations extend over regions of spatial dimension l much larger than the molecular dimensions a ( a/l≪1 ).
(ii) The curvatures of the nematic director field are “soft” ( a∇n≪1 ).
(iii) The $D_{\infty h}$ local symmetry of the molecular ordering is preserved (in the sense that conceivabele deviations therefrom are negligible) under the distortions. This is an essential requirement; it is an important prerequisite for defining the local nematic director n(r) , and thereby its curvatures and fundamental elastic deformations.
(iv) The principal value of the nematic order paramater *S* shows negligible spatial variation in the presence of the elastic distortions.
(v) The changes in mass density, induced by the elastic distortions, are also negligible.

Regarding the symmetry condition (iii), it should be noted that a bend deformation introduces the bend vector **b,** perpendicular to the nematic director **n** (see Figure 1); similarly, a splay deformation introduces the splay vector **s**, tangential to **n**. In addition, a twist deformation introduces a pseudoscalar *t*. These deformations can induce polar (such as $P_{⊥},P_{∥}$ discussed in in the previous section) and chiral biases to the molecular ordering through respective couplings with transverse and longitudinal polar aspects of the molecular structure and/or with the chirality of its conformations. However, the condition a∇n≪1 (see condition (ii) above) implies a|b|≪1 , a|s|≪1 and a|t|≪1 . Therefore, the polarity (along or perpendicular to **n**) that can possibly be induced by the splay or bend deformations has, within the FOt formulation, a necessarily negligible effect on the local symmetry of the molecular ordering and is sometimes referred to as *phantom polarity*. Similarly, a twist deformation would induce a chiral influence on the molecular conformations, whose magnitude is on the order of a|t| or smaller, and therefore negligible.

### 2.3. Spontaneous 1-D Modulations of Nematics within the Frank–Oseen Elasticity Theory

We seek the possible stable states in which one or more of the elastic deformation elements b,s,t have non-zero bulk values in the absence of external fields, surface anchoring, etc. We consider 1-D modulations. This means that, defining the fixed direction of modulation as the *Z*-axis, the director n has a fixed orientation on any plane (*X-Y*) perpendicular to *Z* and can vary only from plane to plane on moving along *Z*, i.e., n(r)=n(Z) . Accordingly, with the notation $\dot{f}\equiv \frac{\partial f}{\partial Z}$ , we have for the fundamental deformation elements the expressions:

(2) $$b=-n_{Z}\left[X{\dot{n}}_{X}+Y{\dot{n}}_{Y}+Z{\dot{n}}_{Z}\right];s={\dot{n}}_{Z}\left[Xn_{X}+Yn_{Y}+Zn_{Z}\right];t=n_{Y}{\dot{n}}_{X}-n_{X}{\dot{n}}_{Y}$$

Note that, for these 1-D elastic modulations, the projections of the bend and splay vectors along the modulation directions differ only by a sign, i.e., $Z\cdot s=-Z\cdot b=n_{Z}{\dot{n}}_{Z}$ .

The deviation of the direction of n from the plane normal Z defines the pseudovector ψ(Z)≡(n(Z)×Z) in each *X-Y* plane. Thus, the modulation along the *Z*-axis can be monitored by the variations of both the magnitude and the direction of $\psi =\left(Xn_{Y}-Yn_{X}\right)$ on moving from one *X-Y* plane to another. Obviously, the unperturbed state (uniform n field) corresponds to constant ψ  , which can be reduced to zero everywhere by choosing n to be along **Z**. 

Proceeding in the framework of the FOt, the free energy is to be formulated in terms of the elastic deformation elements b,s,t , the pseudovector ψ and their couplings, in rotationally invariant combinations compatible with the apolarity symmetry ( n⇔−n ). In addition to the fundamental quadratic contributions in Equation (1), with properly renormalized elastic constants to account for possible flexopolarization effects, the following terms, involving ψ , can be included:

(1) A quadratic contribution associated the magnitude of ψ , i.e., $A\left(\psi \cdot \psi \right)=A(n_{X}^{2}+n_{Y}^{2})$ . As this term accounts for the entropic effects of a finite, uniformly aligned, ψ within each *X-Y* plane, the constant *A* comes with a positive sign.
(2) A scalar coupling of ψ with (∇×n) , namely $B\left[\psi \cdot \left(\nabla \times n\right)\right]=-B\left(Z\cdot b\right)=Bn_{Z}{\dot{n}}_{Z}$
(3) A pseudoscalar coupling, $C^{*}\left(Z\times \psi \right)\cdot \left(\nabla \times n\right)=C^{*}t$ .

There are also quadratic combinations which can be represented by the set of two independent terms $D{\left(Z\cdot b\right)}^{2}=D{\left(n_{Z}{\dot{n}}_{Z}\right)}^{2};E^{*}\left[t\left(Z\cdot b\right)\right]=-E^{*}tn_{Z}{\dot{n}}_{Z}$ .

The sign of the pseudoscalar coupling constants $C^{*}$ and $E^{*}$ is reversed on changing the handedness of twist deformation. Clearly these pseudoscalar constants imply the existence of couplings between some chiral aspect at the microscopic level and the macroscopic twist deformation. Such chiral aspects could originate directly from molecular chirality or from the presence of chirally assembled molecular clusters. The former case covers both intrinsically chiral molecules and statistically achiral flexible molecules adopting chiral conformations. Analogously, chiral as well as achiral molecules could present chiral organization within clusters. Clearly, in all cases involving statistically achiral molecules, domains of opposite twist–handedness are thermodynamically equivalent. The signs of the constants *B* and *D* are not fixed by any obvious physical considerations; in fact, the signs of these terms will be shown not to be critical to the 1-D modulations considered below.

Lastly, there are quadratic invariants of the form ${\left(Z\cdot \nabla (n\cdot Z)\right)}^{2}$ , or ${\left(\nabla (n\cdot Z)\right)}^{2}$ ; in the present case of 1-D modulations their contribution is identical to that of the quadratic splay term, i.e., $∼{\left({\dot{n}}_{Z}\right)}^{2}$ and therefore their net effect is simply to further renormalize the splay elastic constant ${K^{\prime }}_{11}$ into an effective constant ${K^{\prime \prime }}_{11}$ . This effective constant, as well as the renormalized bend ${K^{\prime }}_{33}$ , are assumed to preserve positive signs, as required by thermodynamic stability considerations. Regarding the surface terms in the FOt, the “saddle-splay” contribution, ∇⋅(s+b) , strictly vanishes in this case of 1-D modulated nematics, while the contribution of the other surface term, ∇⋅(s−b) , reduces to $K_{13}\frac{\partial }{\partial Z}\left(n_{Z}{\dot{n}}_{Z}\right)$ .

Collecting all the invariant terms that can be formed from the curvatures of **n**(*Z*) and their couplings with ψ(Z) up to quadratic terms, the following expression for the free energy density is obtained:

(3) $$\begin{array}{l} F(Z)=F_{0}+\frac{1}{2}{K^{\prime \prime }}_{11}s^{2}+\frac{1}{2}K_{22}t^{2}+\frac{1}{2}{K^{\prime }}_{33}b^{2}+K_{13}\frac{\partial }{\partial Z}\left(n_{Z}{\dot{n}}_{Z}\right)\\ \;+A\left(1-n_{Z}^{2}\right)+C^{*}t+Bn_{Z}{\dot{n}}_{Z}+D{\left(n_{Z}{\dot{n}}_{Z}\right)}^{2}-E^{*}tn_{Z}{\dot{n}}_{Z} \end{array}$$

Here $F_{0}$ is the free energy density of the non-modulated (uniform **n**) state. An expression whose bulk part, in the 1-D modulated case, is essentially identical to the above expression (aside from the $K_{13}$ contribution) for the free energy density was obtained in ref [29] based on somewhat different postulates and considerations.

**Figure 2:**
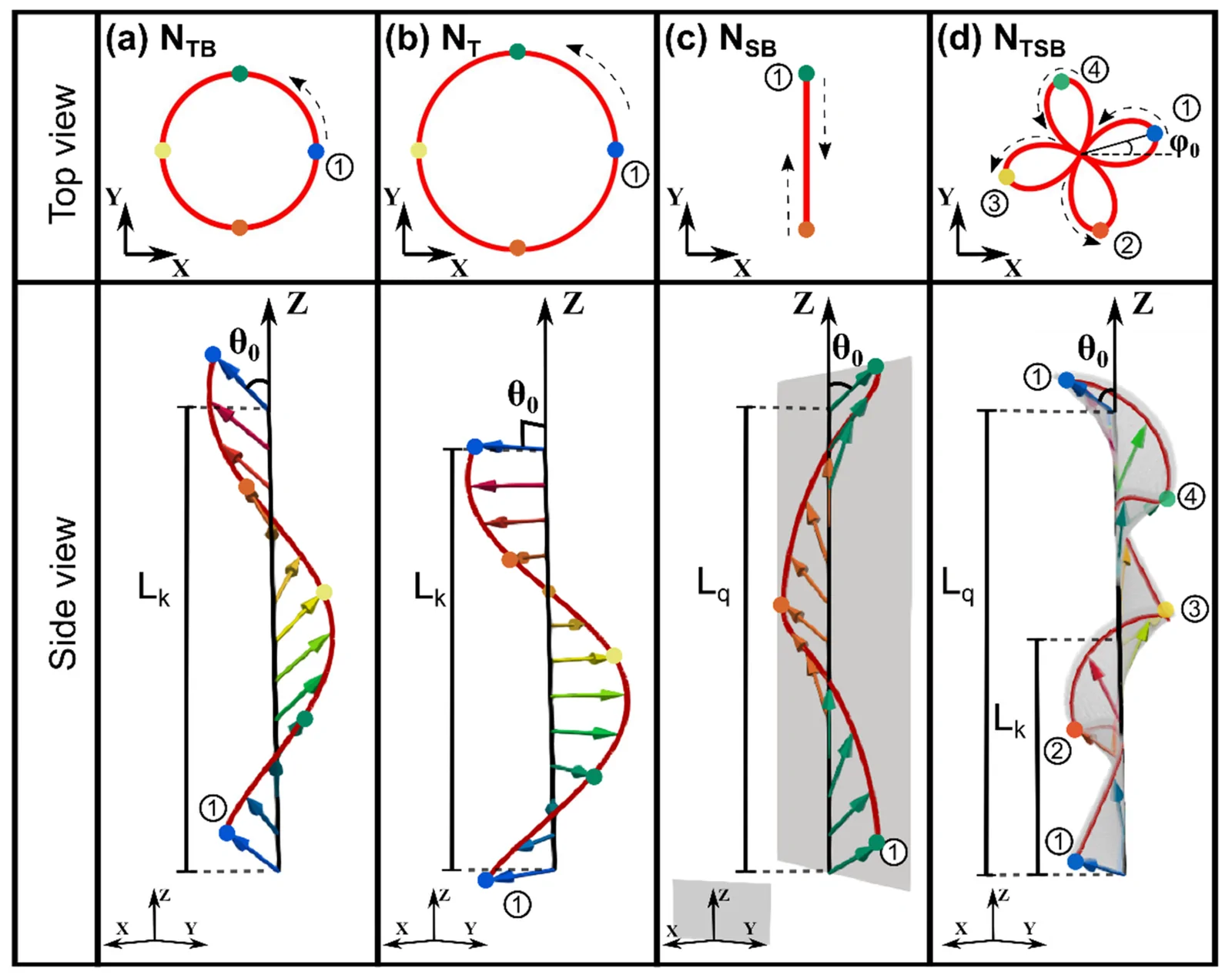
Motifs of 1-D periodically modulated states of a uniaxial nematic elastic continuum; top and side views. The arrows represent the nematic director **n**. *L_k_* and *L_q_* denote, respectively, the repetition lengths of the rotation and vibration modes of the director. (**a**) The twist–bend state, *N_TB_*. The director **n** has a fixed projection, $\cos\theta_{0}$ , along the modulation axis *Z*. (**b**) The pure twist state, *N_T_*. The director **n** is perpendicular to the modulation axis *Z*. (**c**) The splay–bend state, *N_SB_*. The director **n** is shown to remain on the *Y-Z* plane, with its projection along the *Y* axis oscillating with amplitude $\sin\theta_{0}$ . (**d**) A state showing twist, splay and bend deformation (*N_TSB_*) with the oscillation repeat length *L_q_* chosen to be twice the rotation repeat length *L_k_*. The angle $\varphi_{0}$ represents a constant phase difference between the rotation and oscillation modes.

We will limit our attention to periodic modulations of externally unbiased samples, implying that the spatially averaged bend, **b**, and splay, **s**, vectors, over a full repeat length of the modulation, should vanish. This is in accord with the notion of spontaneous symmetry breaking, wherein the symmetries of the ground state (here the fully aligned *N_U_*) are broken locally but preserved globally in the spontaneously deformed states. It is worth noting here that **s** is a local axis of full rotational symmetry (in particular such rotations do not affect the orientation of the local **n**), and **b** is a local *C*_2_ axis preserving the symmetry n⇔−n (i.e., apolarity of the director). Below we consider three possible types of modulations (see Figure 2):

(1) Modulations at constant magnitude of **ψ**; equivalently at constant $n_{Z}$ . The modulation then consists of the periodic rotation of the direction of **ψ**. Such modulations exclude the possibility of splay deformations.
(2) Modulations with **ψ** confined to oscillate along a single fixed axis in the *X-Y* plane. In this case the director reorients periodically while remaining on a fixed plane containing the *Z*-axis. Such modulations exclude twist deformations.
(3) A combination of the previous two modes, i.e., **ψ** confined to oscillate along a single axis whose orientation is rotated on moving along the *Z*-axis.

#### 2.3.1. States of Fixed *n_Z_* and Rotating (n×Z)

In this case $n_{X}=\sin\theta_{0}\cos kZ;\;\;n_{Y}=\sin\theta_{0}sinkZ;\;\;\;n_{Z}=\cos\theta_{0}$ , with $\theta_{0}$ constant. The free energy in Equation (3) reduces to

(4) $$\Delta F\equiv F-F_{0}=\sin^{2}\theta_{0}\left\{A-C^{*}k+\frac{1}{2}k^{2}\left[{K^{\prime }}_{33}+\left(K_{22}-{K^{\prime }}_{33}\right)\sin^{2}\theta_{0}\right]\right\}.$$

Obviously, the state with $\sin^{2}\theta_{0}=0$ corresponds to uniformly aligned director **n** along Z i.e., the *N_U_*. At the other end of the acceptable range of $\sin^{2}\theta_{0}$ , the value $\sin^{2}\theta_{0}=1$ corresponds to **n** perpendicular to *Z*. Minimization of ΔF with respect to k yields, in the case $\sin^{2}\theta_{0}=1$ , the value $k_{T}=C^{*}/K_{22}$ and this “pure-twist” nematic state (*N_T_)* is stable relative to *N_U_* for $\left[{\left(C^{*}\right)}^{2}/2AK_{22}\right]1$ . Accordingly, the wavenumber $k_{T}$ (and therefore the value of the twist pseudoscalar *t*) of the modulation in this purely twisted nematic state has a lower bound given by $k_{T}^{2}2A/K_{22}$ .

The stability of periodic states with intermediate values of $\sin^{2}\theta_{0}$ —these would necessarily exhibit both twist and bend deformations, hence *N_TB_* states—may be explored when ΔF is minimized with respect to both the parameters k and $\sin^{2}\theta_{0}$ . This leads to the requirement $K_{22}{K^{\prime }}_{33}$ for the stability of a *N_TB_* state, with equilibrium values $k_{TB}^{2}=2A/{K^{\prime }}_{33}$ for the modulation wavenumber and ${\sin^{2}\theta_{0}|}_{TB}=\frac{\left|C^{*}\right|\sqrt{{K^{\prime }}_{33}/2A}-{K^{\prime }}_{33}}{K_{22}-{K^{\prime }}_{33}}$ for the “cone angle” formed by the nematic director **n**(*Z*). The latter lies within the acceptable range $0\sin^{2}\theta_{0}1$ provided that ${K^{\prime }}_{33}{\left(C^{*}\right)}^{2}/2AK_{22}^{2}/{K^{\prime }}_{33}$ .

In terms of the dimensionless parameters $g\equiv {\left(C^{*}\right)}^{2}/2AK_{22}$ , $\rho \equiv {K^{\prime }}_{33}/K_{22}$ , and of $k_{0}^{2}\equiv 2A/K_{22}$ , the stability conditions and the respective wavenumbers and cone angles $\theta_{0}$ for the *N_T_* and *N_TB_* states can be summarized as follows:

*N_T_*: stability relative to *N_U_* for g1 ;

modulation wavenumber $k_{T}=k_{0}\sqrt{g}$ ; ${\sin^{2}\theta_{0}|}_{T}=1$

*N_TB_*: stability relative to *N_U_* for ρg1/ρ with ρ1 ;

modulation wavenumber $k_{TΒ}=k_{0}/\sqrt{\rho }$ ; ${\sin^{2}\theta_{0}|}_{TΒ}=\frac{\sqrt{g\rho }-\rho }{1-\rho }$ .

Note that there is a common *g* 1 range over which both the *N_TB_* and *N_T_* solutions exist and are stable relative to the *N_U_*. It is straightforward to show that, in that common range, the *N_TB_* is stable over the *N_T_* due to the condition *ρ* 1.

Clearly, the pseudoscalar coupling parameter $C^{*}$ is essential for the formation of both the *N_T_* and the *N_TB_* states. In physical terms, the existence of some chiral aspect at the molecular level, and the strength of its coupling to the twist elastic deformation is a determining factor for the appearance of these states. Specifically, for the *N_TB_* state, there is no particular requirement regarding the bend elastic constant ${K^{\prime }}_{33}$ other than $0{K^{\prime }}_{33}K_{22}$ . Notably, for the assumed *A* 0, a negative value of ${K^{\prime }}_{33}$ implies physically unacceptable stability conditions ( $k_{TB}^{2}0$ )!

**Figure 3:**
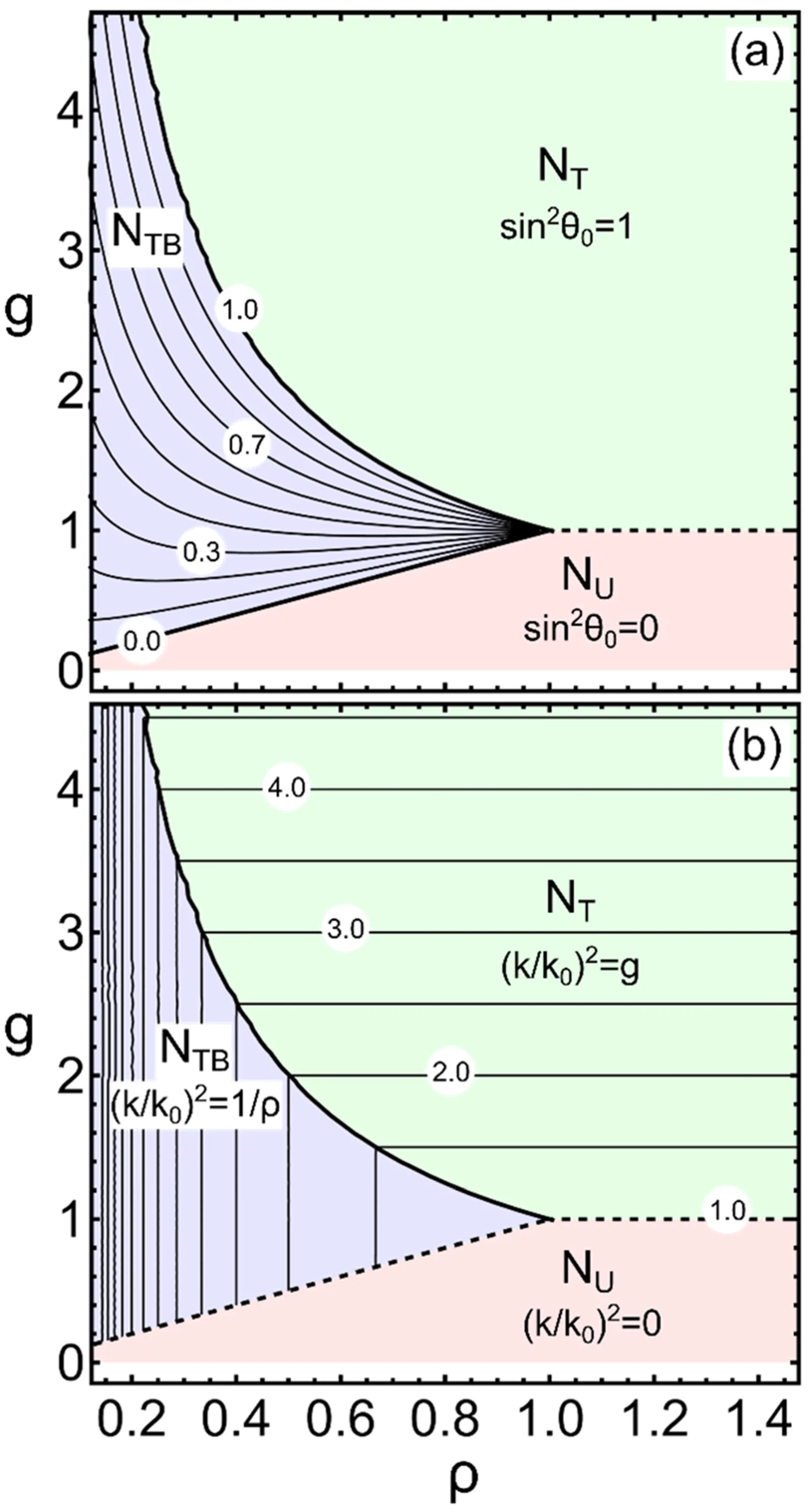
Phase diagrams in the space of the dimensionless parameters $g\left(={C^{*}}^{2}/2AK_{22}\right)$ and $\rho \left(={K^{\prime }}_{33}/K_{22}\right)$ showing the boundaries of states with constant projection of the uniaxial nematic director **n** along the modulation direction Z. For *N_U_* the projection is maximal, n⋅Z=1 ; for *N_T_* it is minimal, n⋅Z=0 and intermediate for the *N_TB_*, $n\cdot Z=\cos\theta_{0}$ . The values of $sin^{2}\theta_{0}$ and of the scaled wavenumber $k/k_{0}$ are indicated on the constant-value contours of (**a**,**b**) respectively.

In Figure 3 we plot the dependence of $\sin^{2}\theta_{0}$ and ${\left(k/k_{0}\right)}^{2}$ as functions of the dimensionless parameter *g* and of the elastic constant ratio *ρ*. One might naively deduce from the relation $k_{TΒ}=k_{0}/\sqrt{\rho }$ that, for finite $k_{0}$ , the wave vector $k_{TB}$ could grow unboundedly large as the ratio $\rho (={K^{\prime }}_{33}/K_{22})$ tends to zero, i.e., as ${K^{\prime }}_{33}\to 0$ . Although the possibility of the renormalized bend elastic constant becoming very small, or even crossing through a null value, is not in principle ruled out, it should be kept in mind that, within the FOt description, the repeat length of the modulation (pitch), $L^{TB}=2\pi /k_{TB}$ , should remain much larger than the molecular dimensions, otherwise the whole continuum approach brakes down (see Section 2.2). In other words, the self-consistency of the FOt restricts the unlimited growth of $k_{TΒ}$ . This remark is particularly relevant to interpretations that glibly attribute molecular length-scale dimensions to the *N_TB_* pitch.

The possibility of an elastically modulated state of constant twist and bend (*N_TB_*) was first demonstrated by R.B. Meyer [15] in the context of the FOt and under the assumption of the presence of a finite polarization **P^0^**. More than two decades later, a twist–bend state was derived starting from the FOt but assuming a negative bend elastic constant, which makes it necessary to include higher order curvatures of the nematic director in the free energy [30]. None of these assumptions are necessary (nor are they made) in the present derivation.

#### 2.3.2. States with **n** Oscillating on a Fixed Plane

Suppose that the *X*, *Y* axes are chosen so that the periodic modulation of the director takes place on the *Y-Z* plane. Assuming a simple harmonic oscillation of repeat length $L_{q}=2\pi /q$ for this “splay–bend” modulation we have

(5) $$n_{X}=0;n_{Y}=\sin\theta_{0}\cos qZ$$

and the free energy density of Equation (3) is expressed as:

(6) $$\begin{array}{l} F_{SB}(Z)-F_{0}=\sin^{2}\theta_{0}\\ \times \left\{\begin{array}{l} \frac{1}{2}{K^{\prime \prime }}_{11}q^{2}\sin^{2}\theta_{0}\frac{\sin^{2}qZ\cos^{2}qZ}{1-{\left|\sin\theta_{0}\right|}^{2}\cos^{2}qZ}+\frac{1}{2}{K^{\prime }}_{33}q^{2}\sin^{2}qZ+K_{13}q^{2}\cos2qZ\\ +A\cos^{2}qZ+\frac{1}{2}Bq\sin2qZ+\frac{1}{4}Dq^{2}\sin^{2}\theta_{0}\sin^{2}2qZ \end{array}\right\} \end{array}$$

Note that the director modulations of Equation (5) lead to vanishing average splay and bend over a repeat length of the modulation. However, the singling out of a plane, in this case defined as the *Y-Z* plane, breaks the global full rotational symmetry. This would imply that particular external stimuli are applied; the respective free energy contribution is not included in Equation (6). 

Unlike the *Z*-independent expression in Equation (4), this form of the free energy density has a periodic *Z*-dependence of repeat length $L_{q}/2$ . Therefore, to look into the stability of this “splay–bend” modulated nematic state throughout the *Z*-range we consider the integrated free energy over a full repeat length of the free energy density. Assuming constant molecular density and degree of ordering, as dictated by the conditions (iv) and (v) in Section 2.2, we have

$$\frac{2}{L_{q}}\int_{0}^{L_{q}/2}F_{SB}(Z)dZ-F_{0}∼{\left|\sin\theta_{0}\right|}^{2}\left(q^{2}C_{1}+A\right).$$

Therefore, the modulated phase cannot be continuously stabilized for any finite q irrespectively of the sign of the composite parameter $C_{1}$ . This does not eliminate the possibility of stabilization of modulated “splay–bend” domains over a fragment of $L_{q}/2$ , in contact with *N_U_* domains. Note that the *K*_13_ and *B* terms do not contribute to the integrated free energy, but they do influence the free energy over fragments of $L_{q}/2$ . The possibility of alternating *N_SB_*−*N_U_* domains will not be further considered here. Different mechanisms for the stabilization of *N_SB_* states are discussed in [31].

#### 2.3.3. States with **n** Oscillating on a Rotating Plane

This can be equivalently viewed as a twist–bend modulation with periodically varying “cone angle”. The latter variation generates a splay deformation and the resulting total modulation can be termed as twist–splay–bend (*N_TSB_*). Such a state, showing simple harmonic *Z*-dependence of the director components can be represented by the following parameterization:

(7) $$n_{X}=n_{⊥}\cos\varphi ,n_{Y}=n_{⊥}\sin\varphi ;n_{Z}=\sqrt{1-n_{⊥}^{2}};\varphi =\varphi_{0}+kZ;n_{⊥}=\sin\theta_{0}\cos qZ$$

For the modulation to be periodic, the wave numbers *q, k* should be integer multiples of a fundamental wavenumber *p*_0_, i.e., $q=l_{q}p_{0}\;\;,\;\;k=l_{k}p_{0}$ , with $l_{q}\;,\;l_{k}=1,2,3...$ . The repeat length of the modulation is then $2\pi /p_{0}$ . Furthermore, the vanishing of the averaged splay and bend vectors over a repeat length of the modulation requires that the sum of the integers $l_{q},l_{k}$ be an odd integer, namely $l_{q}+l_{k}=2l+1$ , with l=1,2,3...

According to Equation (3), the free energy density in this case reduces to:

(8) $$F_{TSB}(Z)=F_{SB}(Z)-C^{*}kn_{⊥}^{2}+\frac{1}{2}k^{2}\sin^{2}\theta_{0}\times \left\{\begin{array}{l} \left[\left(K_{22}-{K^{\prime }}_{33}\right)n_{⊥}^{2}+{K^{\prime }}_{33}\right]\cos^{2}qZ+\\ +E^{*}\left(q/k\right)\sin2qZ \end{array}\right\}.$$

Obviously, for q→0 or for k→0 the above expression tends, respectively, to the expressions in Equation (4) or Equation (6). Below we illustrate the case where the oscillation wavenumber *q* is identified with the fundamental wavenumber $p_{0}$ . This corresponds to setting $l_{q}=1$ , in which case the repeat length of the modulation is $L_{q}=2\pi /q$ and the wave number *k* is an even multiple of *q*, i.e., k=2lq . The periodicity of the free energy density in Equation (8) is in this case $L_{q}/2$ . Therefore, *Z*-integration over the latter length yields:

$$\frac{2}{L_{q}}\int_{0}^{L_{q}/2}F_{SB}(Z)dZ-F_{0}∼\sin^{2}\theta_{0}\left(q^{2}C_{1}+A-C^{*}2lq+l^{2}q^{2}\left[\frac{3}{2}\left(K_{22}-{K^{\prime }}_{33}\right)\sin^{2}\theta_{0}+2{K^{\prime }}_{33}\right]\right)$$

It is apparent from this expression that for given *l*, a continuous *N_SBT_* state can be stabilized for finite wavenumber *q,* under certain conditions for the parameters $C_{1},A,C^{*},K_{22},{K^{\prime }}_{33}$ . However, this stability tends to increase with increasing *l*, indicating that, unless specific boundary conditions favoring a finite *l* are imposed, the most stable state is the one for which l→∞ , therefore q→0 , i.e., the *N_TB_*. Of course, as in the case of the *N_SB_*, stabilization of alternating domains over fragments of the repeat length are not in principle ruled out and in this case the overall stability is influenced also by the terms involving *K*_13_, *B* and *D* together with the free energy density at the interface of the alternating domains.

In summary, for the simple harmonic 1-D elastic modulations considered in this section, only the *N_T_* and *N_TB_* are associated with a *Z*-independent free energy density (compare Equation (4) with Equations (6) and (8)), and these are the only states that can in principle be stable continuously over an arbitrary *Z*-range without the need of particular stabilizing boundary conditions. 

Naturally, in actual samples, the modulation *Z*-range as well as the transverse *X-Y* sample dimensions are finite, implying that the stability will, in any case, be somehow influenced by boundary conditions. This has to be taken into account particularly in relation to the length scale of the modulations: Since the validity of the nematic elasticity FOt is restricted to modulations typically on the order of 1 μm or larger (see condition (i) in Section 2.2), the *X*, *Y* and *Z* dimensions of the sample should be orders of magnitude larger in order for the periodicity along the *Z*-direction to be allowed to develop and, furthermore, in order for the 1-D character of the modulation to avoid inhibition by lateral, boundary-surface-induced deformations. These considerations are of particular relevance to experimental attempts at stabilizing, identifying and characterizing states of truly elastic 1-D spontaneous modulations, such as the *N_TB_* or the *N_SB_.*

## 3. Modulations of Molecular Ordering in Nematics

These are quite distinct from the elastic modulations of the previous section, primarily in that the modulation length-scale is not restricted to macroscopic lengths but can extend down to molecular dimensions. Nanoscale modulations have been long known in smectic liquid crystals; these include the molecular density modulations characteristic of SmA and SmC (synclinic) [32] as well as the combined density and orientational-order modulations typical of the SmC (anticlinic) [33,34] and of a variety of short-pitch, tilted smectics [35]. The nanoscale modulations in the case of smectics are one-dimensional. Analogous density and density-orientation modulations in two dimensions characterize the various columnar phases [32]. Nematics have, by definition, uniform density and therefore only modulations of orientational ordering are applicable. However, until very recently, no such nanoscale modulations were known experimentally, or even conceived of theoretically. Interestingly, the first experimental indications [1,4,36,37] of periodic orientational-order modulations in nematics of achiral molecules were (mistakenly) described in terms of elastic deformations [36], despite the orders of magnitude difference in the length scales involved. Here we outline the description of 1-D molecular order modulations with emphasis on the fundamental differences in the methodology and in the underlying physics *vis a vis* the elastic deformations of the director field **n**(**r**). 

The modulations of molecular order in a nematic phase are directly reflected on the single-molecule distribution function $f_{s}(\omega )$ . This function gives the probability of finding a (generally flexible) molecule of the phase in conformation *s* and orientation *ω* relative to a phase-fixed macroscopic frame *X*, *Y* and *Z*. The orientation is often conveniently described in two steps, with the help of the so called “director frame” in which the distribution of molecular orientations is subject to specific symmetries. Thus the orientation of the molecular axes relative to the director frame is denoted by $\omega_{D}$ , the orientation of the latter relative to the macroscopic frame is denoted by Ω and the distribution function is equivalently written as $f_{s}(\omega_{D};\Omega )$ . In the absence of spatial modulations the director frame has a constant (uniform throughout the spatial extent of the phase) orientation relative to the phase-fixed frame. The two frames can therefore be chosen to coincide and in this case the distribution function is simply $f_{s}(\omega_{D})$ . For a nematic phase presenting 1-D modulation of the molecular ordering, defining *Z* as the modulation axis and *X*, *Y* remaining arbitrary, the orientation Ω depends on the *Z*-coordinate, Ω=Ω(Z) , indicating that the director frame is fixed on any *X-Y* plane and its axes can only change direction on moving along the *Z*-axis. Accordingly, the distribution function in this case is written as $f_{s}(\omega_{D};Z)$ .

To formulate $f_{s}(\omega_{D};Z)$ , one can start from a concrete model for the molecular structure and for the intra-and intermolecular interactions. A closed form for the distribution function is eventually reached in terms of the so called effective potential, defined by $f_{s}(\omega_{D};Z)∼\exp\left(-\bar{V}(\omega_{D};Z)/k_{B}T\right)$ , *via* a statistical mechanical approximation for the evaluation of the average potential energy of a dimer molecule [16] (also of a rigid [38] or flexible [39] solute molecule) as a result of its interaction with the other dimer molecules in the phase. 

**Figure 4:**
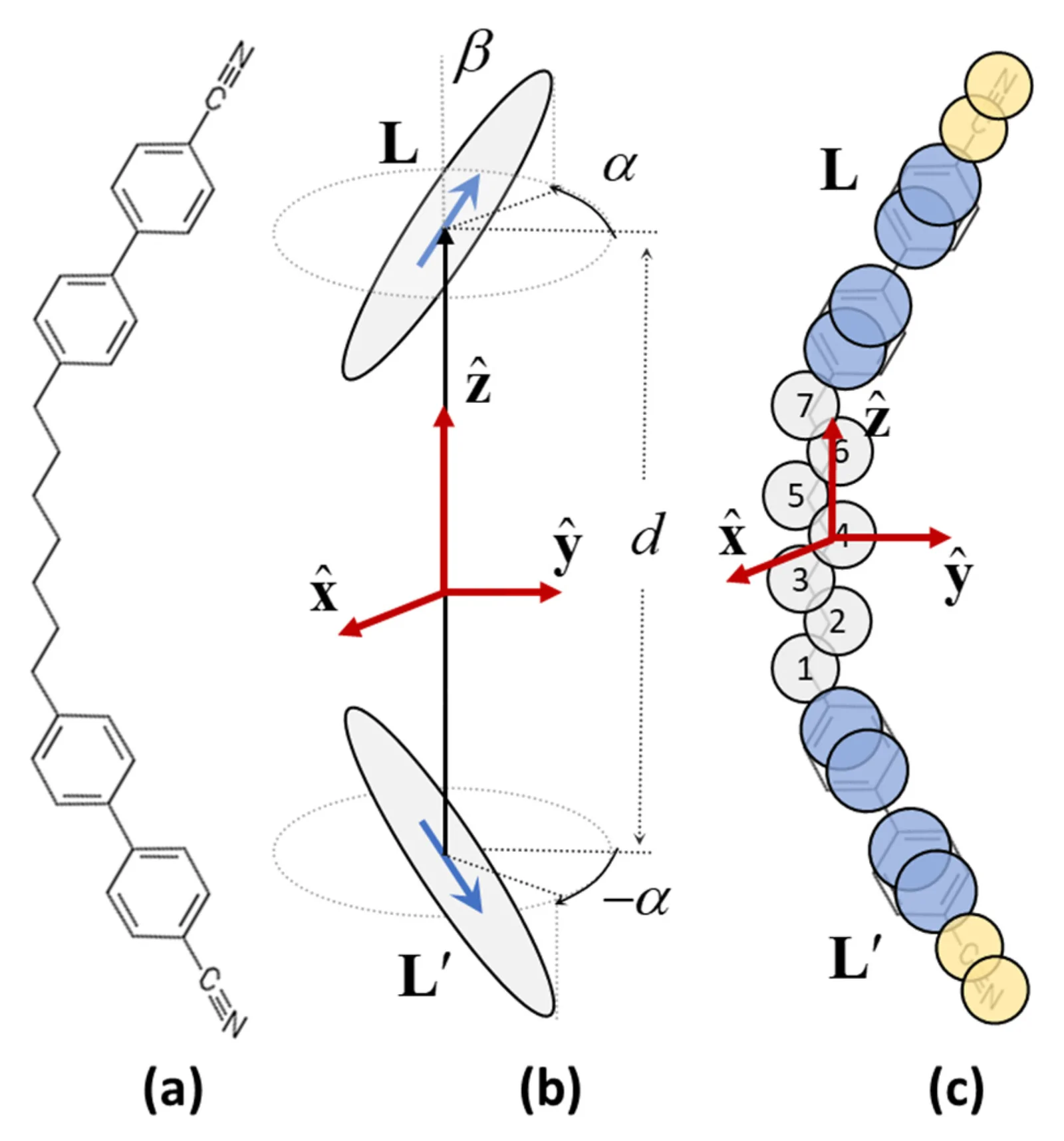
(**a**) Molecular structure of the CB-*7*-CB molecule in the all-trans conformation. (**b**) Molecular model used in the molecular theory of Section 3. The model mimics minimally the architecture of the mesogenic dimer in (**a**), particularly the bent-core structure and statistical a-chirality (equal weight for α and –α angles). (**c**) Spherical united atom representation of the mesogenic dimer in (**a**) as used in the molecular dynamics simulations of Section 4. The model is endowed with the full flexibility of the spacer chain, with conformations generated according to the Ryckaert–Bellemans torsional potential and segmental interactions parameterized as in [40]. The rigid segments **L** and **L’** in (**b**,**c**) denote the mesogenic units of the dimer molecule.

A detailed application of this procedure has been presented [16] in an attempt to rationalize the structure of the low temperature nematic phase (*N_X_*) formed by a variety of LC dimers with bent average structure, the CB-*n*-CB with odd-*n* (see Figure 4a) being a typical representative.

The molecular model used (see Figure 4b for structure and molecular axes assignment) embodies minimally the essential features of this class of symmetric dimers: statistical achirality, two mesogenic units in a bent configuration, elementary flexibility (by allowing for just two chiral conformations of opposite handedness) and *C*_2_ symmetry about a common axis (the **y** axis in Figure 4b) for both conformations. This molecular *C*_2_ axis can show preferential alignment along a polar phase axis **m** that becomes a *C*_2_ symmetry axis of the phase i.e., a “polar director”. The modulation of the molecular ordering consists of the continuous roto-translation of **m** about the modulation axis Z, to which it remains perpendicular, according to

$$m_{X}=\cos\varphi ;m_{Y}=sin\varphi ;m_{Z}=0,\;\mathrm{with}\;\varphi =kZ+\varphi_{0}.$$

The molecular interactions are also modelled in the simplest non-trivial scheme: a mere uniaxial second-rank potential $u(R_{ij})\left(\frac{3}{2}{\left(L_{i}\cdot L_{j}\right)}^{2}-\frac{1}{2}\right)$ for any pair of mesogenic units whose orientations are given by the unit vectors $L_{i},L_{j}$ and their centers are a distance $R_{ij}$ apart. 

Finally, the effective potential is obtained by applying the mean field approximation. Despite the extreme simplicity of the molecular modeling and of the statistical mechanical treatment, the resulting effective potential $\bar{V}(\omega_{D};Z)$ has a rich content as it includes terms promoting the polar ordering (although the interaction among the mesogenic units is strictly apolar) and also the roto-translational modulation of the respective polar director on moving along the *Z*-axis. This is a consequence of a key feature of the otherwise quite primitive molecular modeling: the molecules consist of two individually interacting units separated by a finite (non-vanishing and bounded) distance *d* and maintaining a (“bent-core”) configuration that confers to the overall molecule significant deviation from linearity. The crucial contribution to the effective potential comes from a term [16] of the form

$$∼\left[\left(z\cdot Z\right)\left(y\cdot m\right)+\left(z\cdot m\right)\left(y\cdot Z\right)\right]\times \sin\left(\frac{kd}{2}\left(z\cdot Z\right)\right),$$

where Z is the unit vector along the modulation direction (*Z* axis), m is the polar director (local *C*_2_ axis of the phase), y is the molecular *C*_2_ axis and z is the molecular axis along the line connecting the centers of the two mesogenic units of the molecule (Figure 4b). Note that the scale of the modulation wave number *k* is set by the intramolecular distance *d*. 

For reasonable bend-angles β (0 to π/4), the model yields up to 3 positionally disordered fluid phases, the isotropic (*I*) fluid, the uniaxial nematic (*N_U_*) and the so called polar-twisted nematic (*N_PT_*) in which there is polar order along the director **m**, which roto-translates along the modulation direction *Z* at wavenumber *k* of the order of 1*/d*. The local symmetry of the phase is *C*_2_ while the global symmetry of the phase (i.e., averaged over a large number of repeat lengths of the modulation) is uniaxial about **Z** and apolar. Due to the molecular achirality, domains of opposite twisting sense (i.e., differing in the sign of *k*) are thermodynamically equivalent. The thermodynamic stability of the *N_PT_* phase extends over reasonably broad ranges of the geometrical parameters of the molecular model and, depending on the particular values of these parameters, the *N_PT_* can be obtained, on lowering the temperature, either from the sequence $I\to N_{U}\to N_{PT}$ or directly from the isotropic fluid. The *N_PT_* phase shows very strong polar ordering of the **y**-molecular (*C*_2_) axis along the polar director **m,** quantified by the order parameter P=〈y⋅m〉 . On the contrary, the handedness of the roto-translation modulation appears to induce only marginal shifts (induced molecular achirality) in the balance between molecular conformations of opposite handedness.

**Figure 5:**
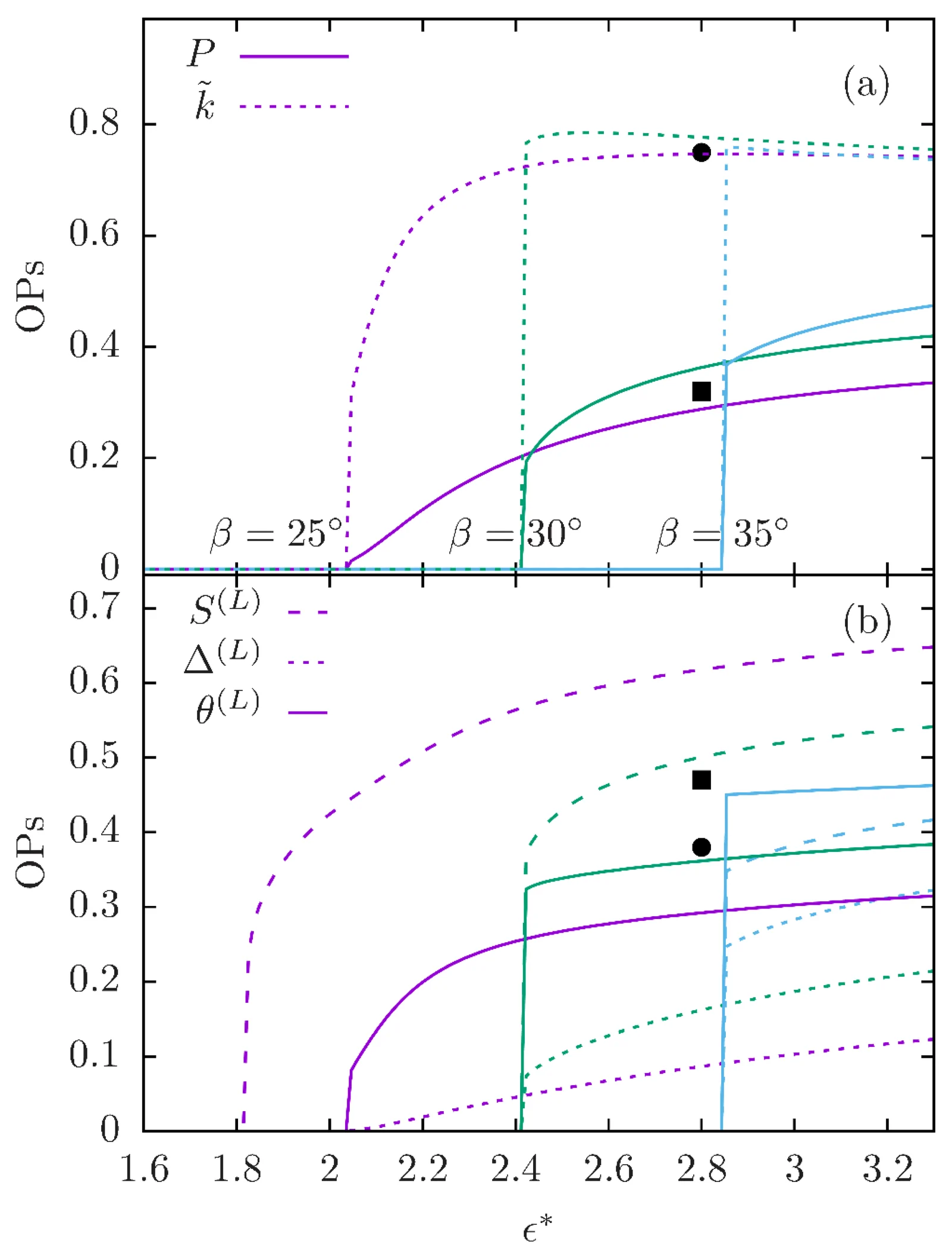
Representative diagrams of molecular theory results for the parameters characterizing the modulated ordering in the *N_PT_* phase as a function of the dimensionless inverse-temperature parameter $\varepsilon^{*}\left(∼1/T\right)$ for three different values of the “bend-angle” β (see Figure 4b) and the torsion angle α held at α=0 . The phase sequence for β=25° is $I\to N_{U}\to N_{PT}$ , with decreasing temperature; for the values β=30°,35° the sequence is $I\to N_{PT}$ . (**a**) Plots of the modulation wavenumber *k* scaled over the molecular size *d* ( $\tilde{k}\equiv kd$ ) and of the polar order parameter P=〈y⋅m〉 , measuring the degree of polar alignment of the *C*_2_ molecular axis **y** along the local polar director **m**. The points indicated by solid symbols are MD results [17] for $\tilde{k}$ (circle) and P (square) calculated at $T=0.965T_{N-N_{X}}$ . (**b**) Plots of (i) the maximal principal value $S^{\left(L\right)}$ of the second rank ordering tensor of the mesogenic units, (ii) the difference $\Delta^{\left(L\right)}$ of the other two principal values (i.e., the biaxiality) of the tensor, (iii) of the angle $\theta^{\left(L\right)}$ (in radians) by which the modulation axis Z is locally rotated about the director **m** to obtain diagonalization of the ordering tensor (equivalently, angle formed between the modulation direction Z and the principal axis of the second rank ordering tensor of the mesogenic unit **L**). As in (**a**), the points indicated by solid symbols are MD results [17] for $S^{\left(L\right)}$ (square) and $\theta^{\left(L\right)}$ (circle).

The temperature dependences of the polar order parameter *P* and of the modulation wave vector *k* (in units of 1/*d*) are shown in Figure 5a for different values of the bend angle *β*. It is apparent that the transition temperature and the phase sequence leading to the *N_TP_* (either directly from the isotropic or *via N_U_*) are sensitive to the angle *β*; above a critical value of *β*, the *N_U_* is eliminated from the stable-phase sequence and the transition takes place directly from the isotropic fluid. Similarly, the polar order parameter *P* is sensitive to the angle *β*, as expected from the sensitivity of the molecular-shape-polarity to variations of that angle. The temperature dependence of *P* shows a gradual or abrupt increase at the onset of the modulated phase, depending on whether the transition is from the *N_U_* or directly from the isotropic fluid. Notably, *k* shows marginal dependence on the angle *β* as well as on the reduced temperature, aside from a rapid or abrupt (in the case of direct transition from the isotropic) rise at the onset of the modulated phase. This is in accord with the notion that the modulation is generated by the molecular packing and therefore *k* is essentially determined by the molecular geometry. The situation here is in marked contrast with the elastic modulation wave number $k_{TB}$ , which is determined by the twist and bend elastic constants (see Section 2.3.1).

It is interesting to examine the ordering of the mesogenic units of the symmetric dimer molecule in the *N_PT_* phase. In marked contrast with the *N_U_*, the mesogenic units show strong polar ordering, $P^{\left(L\right)}\equiv \langle L\cdot m\rangle =P\cos\alpha \sin\beta$ , along **m**. The local quadrupolar ordering is also significantly different: the ordering tensor $S_{AB}^{LL}\equiv \langle \frac{3}{2}L_{A}L_{B}-\frac{1}{2}\delta_{AB}\rangle$ has three different principal values, therefore the ordering is not uniaxial. One of the principal axes of the ordering tensor is the polar director **m**; the other two, denoted by $n^{\left(L\right)}$ and $l^{\left(L\right)}$ , are on the plane perpendicular to m and make angles $\theta^{\left(L\right)}$ and $(\pi /2)-\theta^{\left(L\right)}$ with the modulation axis *Z*. Of course, neither of $n^{\left(L\right)}$ or $l^{\left(L\right)}$ is a symmetry axis of any kind. The angle $\theta^{\left(L\right)}$ , together with the major principal value $S^{\left(L\right)}$ of the ordering tensor, corresponding to the principal axis $n^{\left(L\right)}$ , as well as the difference $\Delta^{\left(L\right)}$ of the other two principal values are plotted in Figure 5b as functions of the reduced temperature for selected values of the angle β . 

**Figure 6:**
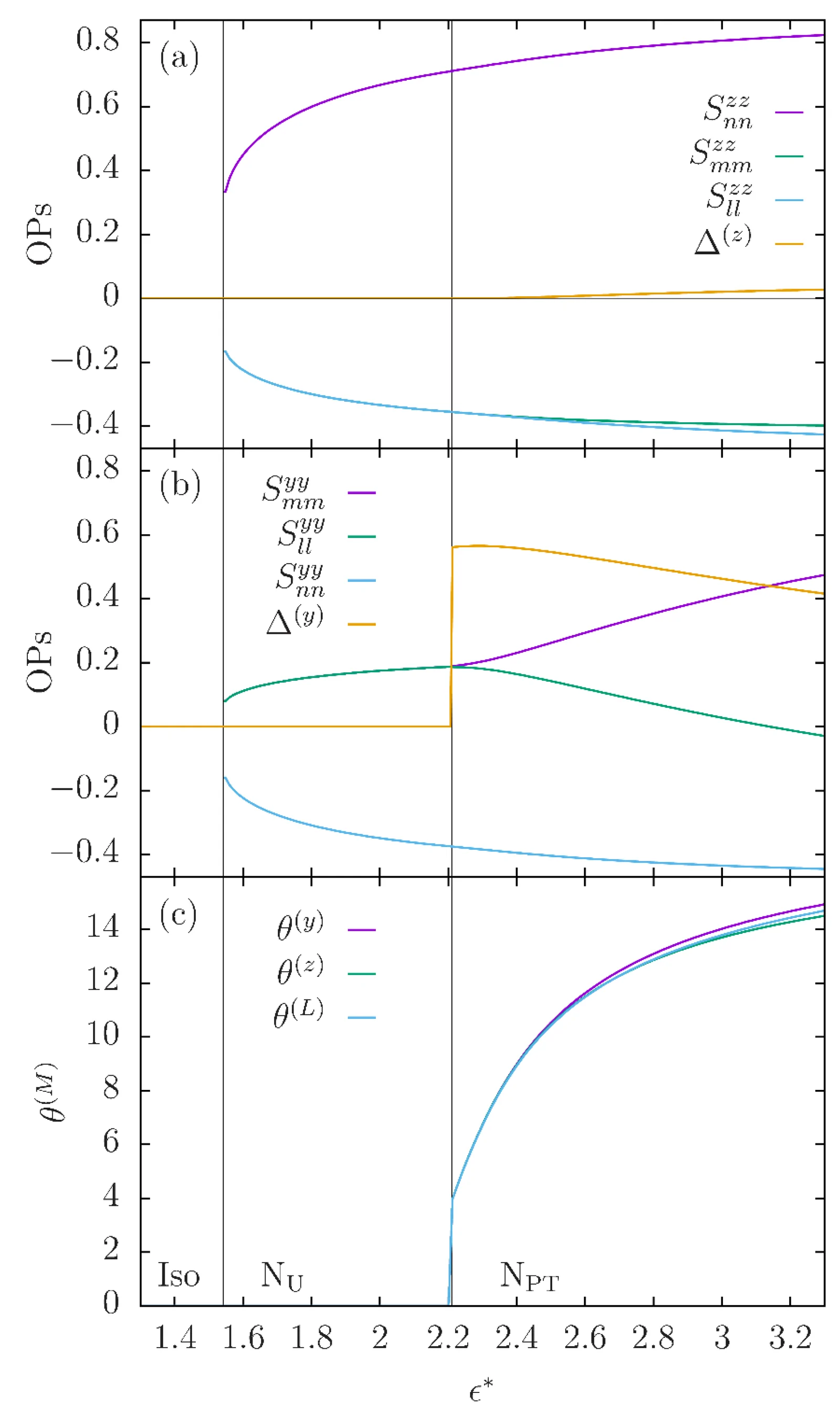
Temperature dependence, calculated from the molecular theory [16] for principal values and rotation angles for the second rank ordering tensors of the *C*_2_ molecular axis **y** and of the molecular axis **z** connecting the centers of the mesogenic units of the model dimer molecule (see Figure 4b). The polar director **m** is, by symmetry, a common principal axis for all the tensors. The other two principal axes, denoted by $n^{\left(M\right)},l^{\left(M\right)}$ , are obtained through a rotation about **m** by an angle $\theta^{\left(M\right)}$ . The plots are for β=25° and α=30° . (**a**) The three principal values of the ordering tensor for the molecular axis **z**. It is seen that the maximal ordering of this axis in the *N_PT_* is along a direction, $n^{\left(z\right)}$ , perpendicular to **m.** The biaxiality (difference the principal values $\Delta^{\left(z\right)}=S_{mm}^{zz}-S_{l^{\left(z\right)}l^{\left(z\right)}}^{zz}$ ) of the ordering sets in below the transition from the N_U_ and remains relatively small throughout the *N_PT_* range. (**b**) Same as in (**a**) for the *C*_2_ axis of the molecule, **y**. Here, however, the maximal ordering of this axis in the *N_PT_* is along **m.** Thus the major principal value is $S_{mm}^{yy}$ and the biaxiality $\Delta^{\left(y\right)}=S_{n^{\left(y\right)}n^{\left(y\right)}}^{yy}-S_{l^{\left(y\right)}l^{\left(y\right)}}^{yy}$ sets in abruptly at the transition and remains large in the *N_PT_*. (**c**) Temperature dependence of the rotation angles $\theta^{\left(M\right)}$ , for the molecular segments *M* = *y*, *z*, and *L*. The differences are not large, but increase with decreasing temperature.

Obviously, the angle $\theta^{\left(L\right)}$ is not to be confused with the angle $\theta_{0}$ of the twist–bend state, nor should the principal axis $n^{\left(L\right)}$ be confused with the nematic director **n** under the respective twist and bend deformation. Whilst $\theta_{0}$ is identical for all molecular segments in the *N_TB_* state, each molecular segment M has its own principal axis frame in the *N_PT_* phase (with the *C*_2_ symmetry axis **m** being common to all such frames) and therefore the angle $\theta^{\left(M\right)}$ is different for different molecular segments. This has direct bearing on observables obtained by NMR measurements in the *N_X_* phase [38,39]. In Figure 6, the temperature dependence of the angles $\theta^{\left(M\right)}$ and the respective major principal values $S^{\left(M\right)}$ are plotted for two molecular axes (see Figure 4b), i.e., for M = y,z , together with the difference $\Delta^{\left(M\right)}$ between the two minor principal values of the second rank ordering tensors for these molecular axes. A clear segment-dependence of both the degree of ordering $S^{\left(M\right)}$ and of the biaxiality $\Delta^{\left(M\right)}$ is apparent from the plots in Figure 6a,b and also from Figure 5b for the mesogenic units (i.e., M = *L*).

**Figure 7:**
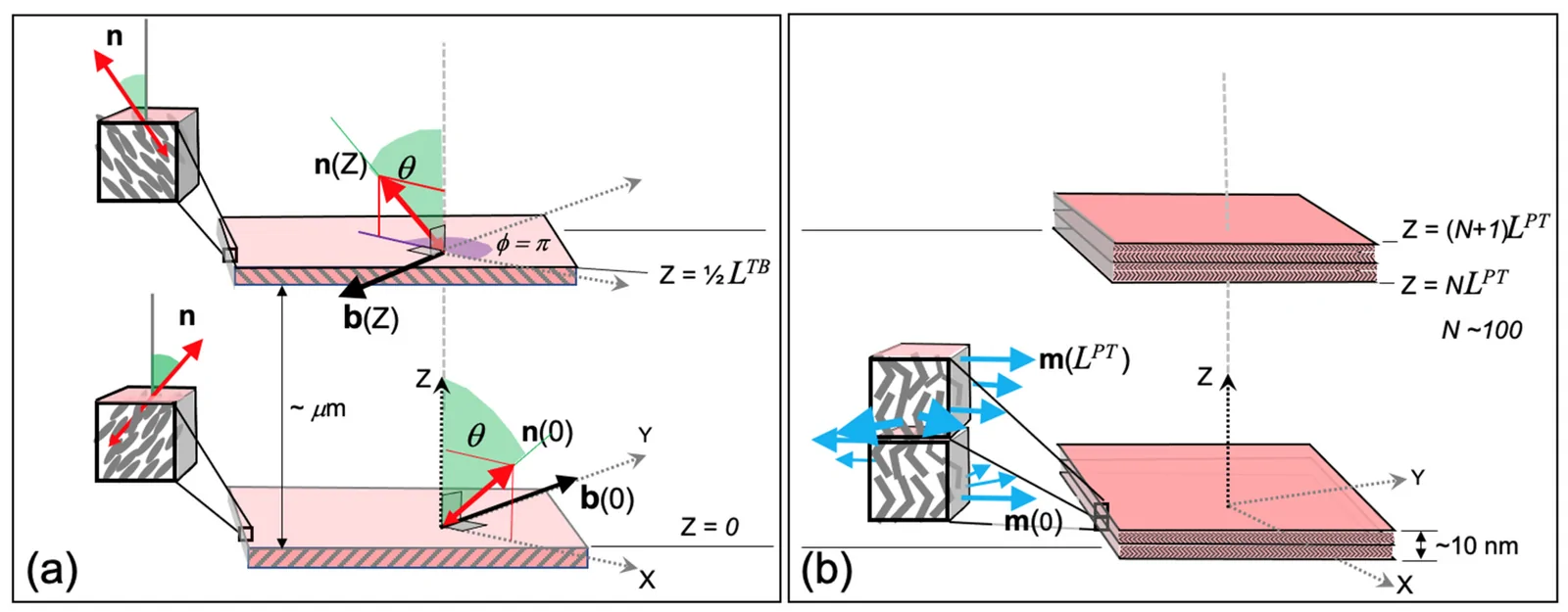
Modulation scales and local molecular ordering in the twist–bend and polar-twisted nematic liquid crystals. Both 1-D modulated phases are partitioned into planar “slabs” stacked perpendicular to the modulation direction (coincident with the *Z*-axis) and of sufficient thickness to define the local director(s). (**a**) The *twist–bend* nematic *N_TB_* has a 1-D elastic modulation of **n** twisting about *Z* with pitch *L^TB^* (~μm*)*; the varying azimuthal angle *φ*(*Z*) is defined by the projection of **n**(*Z*) in the *X*-*Y* plane; within each slab **n**(*Z*) has a uniform direction and maintains a constant projection (cos *θ*) on *Z*. (**b**) The “director” of the *polar twisted* nematic *N_PT_* is the vector **m** (a *C*_2_-axis); it has a uniform polar orientation within sub-planes constituting each slab; **m**(*Z*) undergoes roto-translations about the *Z*-axis on a nanoscopic scale exhibiting a 1-D modulation pitch *L^PT^*(~10 nm). In both (**a**) and (**b**) the cuboid inset dimensions are ~5 nm on edge, large enough to enable the local identifications of **n** and **m**; linear “monomer” and bent-core dimer molecules are indicated by idealized average shapes in the cuboid insets.

In summary, although the ordering modulation in *N_PT_* is parameterized in terms of a wave vector *k* and a variety of deviation angles $\theta^{\left(M\right)}$ , these parameters have distinctly different physical meaning from the parameters *k_TB_* and $\theta_{0}$ that apply to the *N_TB_* state of modulated uniaxial nematic elastic continua. These distinct differences have often been overlooked (as pointed out in [14,41], see also Figure 7 below) resulting in confused pictures of the underlying physics, the length scales and the symmetries of the *N_X_* phase.

## 4. Molecular Dynamics Simulations of Modulated Nematic Ordering

The results regarding the structure and stability of the *N_PT_* phase presented in the previous section need to be tested in at least two respects:

(1) The thermodynamic stability of the *N_PT_* phase, relative to the isotropic fluid and the *N_U_* phase, found over a range of the model parameters, does not exclude the possibility that, over the same parameter range, some other LC phase (e.g., smectic) or even a solid, would be more stable than the *N_PT_*.
(2) How would the stability and the main structural characteristics of the *N_PT_* phase be influenced if the primitive molecular model were enriched by realistic features of the actual molecules, e.g., the CB-*n*-CB dimers of odd *n*, forming the experimental *N_X_* phase? Such features include the extensive flexibility of the spacer chain and the related partition of the molecular interactions into successions of aromatic–aliphatic–aromatic zones which are thought to promote structural microsegregation.

Molecular simulations provide a fairly reliable means of elucidating such points as the stable phase under given conditions is produced by the simulation and the molecular structure can accommodate considerable complexity, as opposed to the molecular theory approach wherein the various phases are predetermined, their relative stability is assessed on the basis of a postulated form of the free energy (in terms of effective molecular potentials and distribution functions) and computational tractability restricts the modeling of the molecular structure and interactions to levels of minimal complexity. 

Indeed, both of the above issues were addressed by molecular simulations using a fairly realistic parameterization (see Figure 4c) of the CB-*7*-CB dimer molecules [17]. The results provided a clear corroboration of the theoretical predictions. Specifically, a second, lower temperature, nematic phase was found to be the stable mode of molecular organization, with polar ordering showing roto-translation modulations on the nanoscale. 

Although the molecular models in Figure 4b,c share in common only very gross features, there is satisfactory quantitative agreement between the results obtained from the molecular theory (model in Figure 4b) and simulation (model in Figure 4c), reflected in the wavenumber values, the polar order parameter and the principal value of the ordering tensor of the mesogenic units. These are shown as singular points in the plots of Figure 5 for comparison with the results obtained from the molecular theory. The scaling of the simulation wavenumber *k* is done with respect to the conformationally averaged distance 〈d〉 between the centers of the mesogenic units of the dimer molecule, i.e $\tilde{k}=k\langle d\rangle$ . The polar order parameter *P* is obtained from the simulations as the averaged projection of the molecular **y**-axis of Figure 4c along the local director **m**. It is apparent from Figure 5 that not only the orders of magnitude but also the numerical values obtained from the MD simulations for $\tilde{k},P,S^{\left(L\right)},\theta^{\left(L\right)}$ are remarkably close to the respective values obtained from the molecular theory calculations based on the minimal molecular model in Figure 4b.

## 5. Discussion

The discrepancies resulting from identifying the *N_X_* phase with the *N_TB_* model were pointed out in a recent paper, *The twist bend nematic: a case of mistaken identity* [14]. Since that publication two additional papers [18,42] appeared opposing the misidentification critique: one paper [42] was a direct rebuttal of [14]; a detailed reply to that rebuttal was given in [41]). Briefly, the authors of the rebuttal—apparently realizing some of the incompatibilities of *N_X_* with elasticity models—have chosen (perhaps as a way of salvaging their initial identification of *N_X_* with *N_TB_*) to broaden the notion of the twist–bend nematic. Thus, they directly advocate that there is no unique *N_TB_* model but an evolving cluster of different *N_TB_* models, not necessarily based on elasticity, into which even the *N_PT_* model is subsumed. In essence, the authors trivialize the question of the structure and underlying physics operative in the *N_X_* to mere nomenclature: The *N_X_* is defined as *N_TB_*, therefore, whatever it takes to describe correctly the *N_X_* is by definition included in their evolving cluster of *N_TB_* models.

The second paper [18] is, in that respect, a few steps behind the cluster advocates as its authors insist on using the idea of elasticity-driven modulations in order to interpret simulation results that, nevertheless, provide clear evidence of polar molecular ordering accompanied by 1-D nanometer spatial modulation of the direction of that ordering. Their modeling mixes macroscopic continuum concepts with local molecular ordering thereby generating inconsistencies in both the length scales and the symmetries on those length scales. In fact, none of the conditions for the applicability of the FOt (see Section 2.1) are valid in their theoretical modeling and simulations. Moreover, their simulation data prove directly that the modulations have nothing to do with elasticity and are generated by molecular organization at the nanoscale. The experimentally determined modulation in the *N_X_* phase is two or more orders of magnitude below the length scale for the applicability of the FOt of elasticity, thus pointing directly to a different underlying physical mechanism. Moreover, the scale difference is reflected in the magnitude of the bend-induced polarity in the *N_TB_* (estimated $\langle P\rangle \sim 10^{-4}$ ) compared to the polarity of the molecular packing (order parameter $\langle P\rangle \sim 10^{-1}$ ) predicted by the *N_PT_* model (Figure 5a) and directly evident from the simulations of [18] and those using more detailed molecular models [17]. Lastly, classifying LCs according to their symmetries obviates the terminology “twist–bend”, “splay–bend”, etc., for the simulated phases in [18] since those classifications presuppose local *D_∞h_* symmetry. But such symmetry is not supported by their simulations, all of which only exhibit lower local symmetry, e.g., *C*_2_.

Aside from the length-scale and symmetry discrepancies, the mix up of continuum with microscopic concepts leads to flawed conclusions on well-established matters. For example, what are termed in [18] as twist–splay–bend phases are subsequently argued to be smectics. However, the only smectic phase wherein the orientational order of the molecules can be described solely in terms of the **n** director is the smectic A phase, and therein the twist deformation of **n** is strictly forbidden (see [21]). Another example is the presentation in [18] of a collinearity relation, between the bend vector **b** of the nematic director **n** and the polarity vector **m**, as proof that phase modulation is driven by a presumed coupling between polar order and bend deformations. Aside from the symmetry incompatibility, this relation reduces to a mere geometrical identity in the context of local *C*_2_ symmetry [16]. Accordingly, when supported by the data, this relation is a clear indication that the medium does have polar order along a single director **m**, which precludes any possibility of $D_{\infty h}$ and concomitantly a nematic director **n** (or bend deformations thereof).

Of course, we do not adopt the view of an evolving “cluster of *N_TB_* models” nor do we place any credibility on shrinking the models based on elastic deformations (e.g., the twist and bend of uniaxial nematics) down to the nanoscale. The length-scale, structure, and symmetry differences between the elasticity-based *N_TB_* and the molecular packing-based *N_PT_* models are enormous. These are graphically summarized in Figure 7.

The *N_TB_* and *N_PT_* models share two common features: (i) both phases are positionally disordered (no density modulation, i.e., the phases are nematic) and (ii) both present a 1-D modulation of the orientational order, and this introduces a fundamental wave number *k*. However, the respective values for this wave number, $k_{TΒ}$ and $k_{PT}$ , differ by orders of magnitude, reflecting the obvious fact that the corresponding modulations stem from entirely different physical mechanisms. It is precisely for this reason that the wave numbers show, in addition to their vastly different magnitudes, markedly different variations with the thermodynamic parameters as depicted in Figure 3b and Figure 5a.

The main differences between the *N_PT_* and the *N_TB_* models are summarized in Table 1. It is apparent from Figure 7 and Table 1 that the structures of the *N_TB_* and *N_PT_* are mutually exclusive, leaving no room for physical consistency in descriptions which arbitrarily combine features from both macro- and microscopic regimes to produce hybrid interpretations.

## 6. Concluding Remarks

Molecular shape and local packing ultimately determine the nature of the long range supramolecular organization found in LCs, and when new molecular shapes are explored, unexpected phases are discovered [2]. This was the case when the *N_X_* phase of odd-*n*-linked CB-*n*-CB dimers was discovered in 1991 [43]. Two decades later, that *N_X_* phase was branded *N_TB_*, despite the incongruency of modulation length scales—continuum versus molecular. One might reasonably ask, “on what length scales might continuum descriptions of soft matter apply?” A minimal answer for fluid phases would be, “At scales larger than those showing structure in its radial distribution function”. For simple liquids that distance is multiple molecular dimensions. The experimental helical pitch exhibited by the *N_X_* phase of the CB-*n*-CB dimers (*L^PT^* ~8 nm) [37,44,45,46] is only slightly larger than twice the dimer length (~3 nm), clearly too small for a continuum description to apply. That tight pitch is predicted, however, by the molecular theory of the polar twisted nematic phase [16]. It remains to be confirmed how a putative conventional uniaxial nematic *N_U_*—the high-temperature, apparently uniaxial phase above the lower temperature *N_X_* exhibited by nonlinear mesogens—could transform directly into the nanometer-scale, modulated structure in the *N_X_*. Interestingly, there are experimental indications [39] supporting the idea that the low temperature *N_X_* phase of the CB-*n*-CB is in fact not obtained from a *N_U_* phase on lowering the temperature but from a phase consisting of fragments of *N_X_*-like aggregates (cybotactic groups) of opposite handedness exhibiting global uniaxial symmetry and achirality on the NMR time-scale. The transition to the *N_X_* phase in that case proceeds via the self-assembly of aggregates of the same handedness to form macroscopic *N_X_* domains. 

Despite the large disparity between length scales and phase symmetries, the elastic deformations of **n** in the continuum approximation (macroscopic) are often mistakenly combined in the literature [14] with polar molecular ordering and molecular-length-scale modulations (microscopic). The continuum framework of Frank–Oseen theory is “by construction” restricted to: (i) uniaxial and apolar symmetry of the local molecular ordering and, (ii) modulation lengths of the local ordering that are much larger than molecular dimensions. Molecular theory suggests, and molecular dynamics simulations prove directly, that the modulations presented by the *N_X_* phase have nothing to do with elasticity and are generated by molecular organization at the nanoscale, i.e., that bent-core molecules exhibit a genuinely different nematic state and not a spontaneous elastic deformation of the conventional *N_U_* phase. 

Lastly, regarding the possibility of preparing and identifying experimentally a true *N_TB_* state (or, for that matter, any of the 1-D elastically modulated states of the uniaxial nematic phase, such as the *N_SB_*, *N_STB_*, alternating, stabilized by particular boundary conditions), consider the implications of the analysis in Section 2: Therein it is suggested that, aside from exploring a regime of an entirely different length-scale in which such states should be looked for, there are a number of factors that need to be combined favorably in order to achieve thermodynamic stability. Perhaps this is, at least part of, the explanation for the elusiveness of these elasticity-mediated states, despite a half-century-long search for these theoretically-predicted, macroscopically-modulated phases.

Although the present discussion focused on the inappropriateness of using an elasticity-based *N_TB_* model to account for the experimentally-established fundamental properties of the *N_X_* phase, similar length-scale and symmetry considerations apply to other dubious identifications [18,19,47] of nanoscale-modulated nematics, i.e., the “splay–bend” nematic, *N_SB_*. In summary, as is generally the case in condensed matter, macroscopic physical models break down at the nanoscale.

## Figures and Tables

**Table 1:** Physical characteristics that differentiate the *N_TB_* model from the *N_TP_* model.

General Aspects	Attributes	*N_TB_*	*N_PT_*
Local Symmetry	Symmetry Group	*D_∞h_*	*C*_2_
	Symmetry Axis	Nematic Director n(↔−n)	Polar Director m(↔−m)
Spatial Modulation (one dimensional)	Type	Twist Bend of **n**	Roto-translation of **m**
	Length Scale	Macroscopic *L^TB^*~μm	Molecular *L^PT^*~10 nm
	Physical Origin	Spontaneous Elastic Deformations of **n**	Polar Molecular Packing along **m**

## Data Availability

Data Availability Statement: Data can be available upon request from the authors.
